# Promoting Small Business Support of Youth Physical Activity in Low-Income, Minority Neighborhoods: Protocol for a Randomized Controlled Trial

**DOI:** 10.2196/13141

**Published:** 2019-07-30

**Authors:** Richard Robert Suminski Jr, Shannon Robson, Jennie Turner, Eric Plautz

**Affiliations:** 1 Center for Innovative Health Research Department of Behavioral Health and Nutrition University of Delaware Newark, DE United States

**Keywords:** health promotion, public health, exercise, children, ethnic groups

## Abstract

**Background:**

An unacceptably high percentage of our nation’s low-income, minority youth (age<18 years) are not regularly physically active. One reason for this could be their lack of access to quality youth physical activity opportunities (YPAOs). Our previous research found that small businesses (<500 employees), which represent over 99.64% (27.9/28.0 million businesses in United States) of all employers, are powerful resources for creating and improving YPAOs. In accordance with the socioecological model and established philanthropic principles, we developed an alpha version of an intervention (alpha-i) for increasing small businesses’ involvement with YPAOs.

**Objective:**

The aims of this proposed study are to (1) create a beta version (beta-i) of the intervention and (2) conduct a pilot study of its impact on small business support for YPAOs and YPAO utilization by the youth in low-income, minority neighborhoods.

**Methods:**

The alpha-i will be refined using information from focus groups and surveys conducted with small business owners and managers, YPAO providers, and parents and guardians of the youths from low-income, predominantly minority neighborhoods. A cluster randomized controlled trial will then be conducted for 1 year to examine the effects of the refined intervention (beta-i) on small business support for YPAOs in 10 low-income, minority neighborhoods. The control group of neighborhoods (n=10) will be provided with a standard practice intervention. The primary outcome for aim 2 will be the percentage of small businesses not supporting YPAOs at baseline that subsequently provide support for YPAOs at follow-up. We also will consider the US dollar equivalent of all types of support (monetary, goods/services, and time) donated for YPAOs by small businesses. In addition, we will examine the impact of the increased small business support for YPAOs on YPAO utilization by the youth.

**Results:**

As of May 1, 2019, all YPAOs and small businesses in the study neighborhoods have been identified, and surveys have begun with these groups. In addition, 9 focus groups were completed, and the data have been transcribed. We anticipate that manuscripts regarding these aspects of the study will be submitted in fall 2019.

**Conclusions:**

The proposed study is significant because it will provide evidence that an easily replicated approach can be used to increase small business support for YPAOs and that this support results in greater use of the YPAOs by youth. A logical next step will be to determine if YPAO changes resulting from increased small business support positively influence youth physical activity levels.

**ClinicalTrial:**

ClinicalTrials.gov NCT03936582; https://clinicaltrials.gov/ct2/show/NCT03936582.

**International Registered Report Identifier (IRRID):**

DERR1-10.2196/13141

## Introduction

### Background

Health benefits can be gained by the youths (age<18 years) who regularly participate in physical activity; for example, physical activity reduces the incidence of overweight, obesity, and chronic diseases [1-4]. Unfortunately, many youths fail to engage in adequate levels of physical activity. National data show that 3 out of 4 youths (aged 12-15 years) do not meet physical activity guidelines for moderate to vigorous physical activity (MVPA), and 7.6% are not involved in MVPA at all [5]. The problem is even more glaring for the minority youth. The 2013 Youth Risk Behavior Surveillance survey indicated that 21.5% of minority youth, compared with 12.5% of nonminority youth, were performing less than the recommended 60 min per week of MVPA [6]. In 1995, similar findings were reported—the minority youth participated in less MVPA than their nonminority peers [7]. This persistent difference has undoubtedly contributed to the disproportionately high burden of disease currently seen in the low-income, minority youth. Recent estimates indicate that 8.0% of African American children (aged 2-19 years) are considered severely obese versus only 3.9% of white children and the incidence of type 2 diabetes is a staggering 5 times higher [8-10]. Given their typically poor outcomes, it is unlikely that school-based physical education (PE) or physical activity interventions conducted in isolation (ie, individual level) will have a sufficient positive impact on physical activity [6,11]. Although over half of the high school youth are enrolled in PE, only 29% attend class on a daily basis and less than half are actually physically active during class [12]. Clearly, discovering innovative ways to enable and encourage the low-income, minority youth to be more physically active is a public health priority, requiring novel ideas and sustainable solutions.

The socioecological model has been used to explain why the youth are not more physically active. Briefly, the model suggests physical activity behavior is determined by intrapersonal (eg, self-motivation), interpersonal (eg, social support), and environmental (eg, availability of programs) factors [13]. Although intrapersonal and interpersonal factors have been studied extensively, they have not been shown to adequately explain youth physical activity or provide a basis for the development of effective interventions for the youth [14]. On the other hand, the evidence supporting a role of the environmental factors has grown exponentially in recent years [15-18]. Community sprawl, safety concerns, pedestrian-unfriendly street designs, and increases in sedentary activities (eg, television), among others, have a negative effect on youth physical activity and fitness [19-23].

Perhaps the strongest and the most influential environmental determinants of youth physical activity are the availability and adequacy of youth physical activity opportunities (YPAOs). They have been defined as programs and places available to the youth with components/amenities that typically require/involve physical activity [24-28]. A park with sports fields or playgrounds or a dance class at a community center are good examples. Significant associations have been found between youth physical activity and access to affordable YPAOs, and lack of YPAOs has been cited by the youth as a major barrier to being active [24,29-35]. The quality of the YPAOs (eg, staff training and equipment) also plays a role and has been shown to be as vital for increasing youth physical activity as having access to YPAOs [21,36,37-40]. Health-related fitness, including body fatness, has been associated with YPAOs, and the youth involved with YPAOs learn sportsmanship, acquire new skills, improve social skills, and are more likely to participate in physical activity as adults [15,41-45].

YPAOs assume added importance if they are in one’s local environment or neighborhood. Walking by the youth (aged 5-20 years) is positively associated with having access to nearby (<1 km distance from home) recreation or open spaces [46]. Middle school children engage in more physical activity if they have available after-school programs and high-quality local facilities near home [25]. Children provided with a safe schoolyard in their neighborhood become more physically active than children not granted such an amenity [21]. Others have shown that recreational facilities closer to home are more likely to be used than facilities located elsewhere and that local neighborhood characteristics, especially the presence of trails and places for physical activity, play a role in leisure activity patterns [24,26,47]. The presence of YPAOs varies between neighborhoods, with economically disadvantaged and minority neighborhoods having significantly fewer YPAOs than more affluent neighborhoods [48-52]. This deficiency, along with concerns about transportation and YPAO expenses, is expressed significantly more often by the parents of the minority youth than the parents of the nonminority youth [31]. Few YPAOs and associated expenses have been cited as critical barriers to reversing physical inactivity among the low-income, minority youth [53-56].

Small businesses (<500 employees) represent 99.64% (27.9/28.0 million) of all businesses operating in the United States. Total revenues typically exceed US $1 trillion annually and over half a million new businesses start each month [57]. More than one-fifth of small businesses are minority-owned with revenues totaling nearly US $700 billion [58]. Established philanthropic principles have been used to explain behaviors of different types of entities including small businesses [59-66]. Event sponsorship or sponsorship marketing refers to supporting various types of initiatives ranging from educational partnerships to YPAOs [59]. Small businesses heavily engage in event sponsorship compared with large businesses and they prefer to contribute to events connected to their local neighborhood [60-62,64]. It allows them to reach their target market more efficiently, expose their product/service directly to the market, give back to the neighborhood that supports them, and present an image of a socially responsible organization [63]. These actions can lead to increased consumer support and ultimately greater revenue [65,66]. Not surprisingly, when small businesses decide to support an initiative, they tend to sustain that support [67].

The strong preference by small businesses for sponsoring local programs sets up a powerful force that, if utilized effectively, would have a dramatic and lasting impact on the quantity and quality of YPAOs and, ultimately, physical activity and health in low-income, minority neighborhoods. Supporting this contention are findings from our preliminary study showing that although a majority of small businesses do not currently support YPAOs (~60%), a large percentage (88%) of these non-YPAO supporters believe they should [67,68].

### Objectives

In accordance with the socioecological model and established philanthropic principles, we developed an alpha version of an intervention (alpha-i) for increasing small businesses’ involvement with YPAOs. We are now poised to create a beta version (beta-i) and conduct a pilot study of its *impact* on small business support for YPAOs and *YPAO utilization by the youth* in low-income, minority neighborhoods. To meet this objective, we will complete the following specific aims and address the specified hypotheses.

- *Aim 1:* Refine alpha-i components by conducting focus groups with small business owners, YPAO providers, and parents and guardians of the youth from low-income, predominantly minority neighborhoods. Results of the qualitative analysis will inform final tailoring of the intervention to create the beta-i that will be tested in aim 2.

- *Aim 2:* Determine the effect of the beta-i on small business support for YPAOs by conducting a cluster randomized controlled trial with randomization at the neighborhood level. The intervention neighborhoods (n=10) will receive the beta-i, whereas the control neighborhoods (n=10) will be provided a standard practice intervention for a period of 1 year.

- *Hypothesis 1:* The beta-i will result in a significantly greater increase in the percentage of small businesses providing support (eg, monetary donations) for YPAOs than a standard practice intervention.

- *Hypothesis 2:* The US dollar equivalent of all types of support (monetary, goods/services, and time) donated for YPAOs by small businesses exposed to beta-i will be greater than that donated by small businesses exposed to the standard practice intervention.

- *Aim 3:* Examine the impact of the increased small business support for YPAOs on YPAO utilization by the youth. The primary outcome will be the percent change in the number of youths participating in YPAOs from baseline to follow-up.

- *Hypothesis 3:* The percent increase in youth participants from baseline to follow-up will be significantly greater at YPAOs in the treatment neighborhoods receiving support from small businesses than at YPAOs in the control neighborhoods.

## Methods

### Procedures for Aim 1

The timeline of activities completed in aim 1 is given in Multimedia Appendix 1. The first set of activities involved identifying the study neighborhoods and the small businesses and YPAOs in these neighborhoods. Next, focus groups were conducted, and a local advisory board was formed. The focus group data were used by the local advisory board to refine the alpha components of the intervention we previously developed to create a beta version that was tested in aim 2 (Multimedia Appendix 2 [69-76]).

#### Study Neighborhood Identification

We used a multistep process recommended for use in urban health research to identify 27 distinct neighborhoods in New Castle, Delaware (mainly Wilmington, the largest city in the state) meeting our inclusion criteria requiring a minority concentration greater than 50%, median household yearly income in the lower third of all neighborhoods in these areas, and a land use mix that is at least 30% residential and at least 15% retail/commercial [77]. From the pool of 27 neighborhoods meeting our inclusion criteria, 20 were randomly selected to participate in the pilot study of the beta-i. Pairs of neighborhoods separated by at least 0.5 miles constituted the randomization unit with 1 randomly assigned to the treatment and the other to the control (see *Interventions* section later in this paper for a description of the study arms). This helped reduce the risk of contamination.

#### Small Businesses and Youth Physical Activity Opportunities Identification

During November and December 2018, we identified all small businesses and YPAOs in the study neighborhoods using various approaches and sources we had used in previous studies (registries, internet/phone books, media, community tours, and community members) [52,67,68,78]. YPAOs were defined as programs and places available to the youth with components and amenities that typically involved physical activity [24-28,52,68]. Some examples of YPAOs we found were playgrounds, ballfields/courts, classes, sports leagues, and various types of structures (eg, jungle gym). These YPAOs were located mainly at parks, churches, and for-profit businesses, which is consistent with previous research [52,79]. We included school components (eg, athletics) because these are important YPAOs [80]. YPAOs not available to the public (eg, worksite exercise programs) or those not designed primarily for physical activity (eg, sidewalks/streets and stairs) were not included. A tracking system developed in our previous study will be used to detect new YPAOs that emerge and dissolve during the intervention [78]. The system involves monitoring local media and internet sources, canvassing neighborhoods, and obtaining feedback from YPAO providers. Follow-up procedures (eg, phone calls) will be carried out to confirm if a YPAO is actually new and baseline data on existing YPAOs will be referenced to quantify the emergence of new ones. The information obtained on YPAOs was and will be carefully reviewed to eliminate duplication.

#### Focus Groups

Focus groups allowed us to obtain unique perspectives from neighborhood stakeholders, which were used to refine alpha-i components to better meet the needs/resources of the treatment neighborhoods. Participants were asked to consider components, provide recommendations, and suggest modifications. We specifically looked for input on our strategy for increasing support for YPAOs, how to efficiently handle administrative tasks, promoting YPAO provider fund use, ways to reduce barriers to using YPAOs, and long-term sustainability.

Between January and March 2019, we conducted 9 focus groups and analyzed the data. Each focus group was comprised 6 to 8 members recruited from the 27 low-income, minority neighborhoods that had met our inclusion criteria as outlined above. Participation was solicited from small business owners (3 focus groups), YPAO providers (3 focus groups), and parents and guardians of the youth (3 focus groups), using our personal contacts, referrals, flyers, and ads in local publications. Incentives (eg, food/drink, $20 gift card) were used to encourage participation and all group sessions were held in one of the study neighborhoods. The number of groups we used was adequate for ensuring saturation, providing us with a comprehensive picture of the domains and helping achieve focus group goals [81,82].

Each focus group lasted about 90 min and was moderated by a research team member with extensive training in qualitative methodology. The moderator was assisted by a second scientist who documented focus group proceedings and other process data (eg, nonverbal behaviors) [83]. Using a focus group guide developed for this project, the moderator walked members through the topics to be covered and used probes to clarify select responses or solicit more detailed information [83]. Members were given a chance to ask questions to ensure they understood the process. At the end of each focus group, the team debriefed by reviewing notes and discussing particularly relevant areas (eg, presence of domineering members) [84,85]. Insights from the debriefing sessions were used to enhance the quality of the data by providing an explanation for areas that seemed ambiguous after all data were transcribed and coded. Each session was audiotaped with 2 electronic digital recorders for later transcription.

#### Preparation for Aim 2

The final activities that were completed in aim 1 involved the refinement of alpha-i components by the local advisory board, mainly using focus group and survey outcomes, and the formation of the beta-i. We also prepared for baseline data acquisition and developed the tracking mechanism that will be used to monitor donations to the fund and the distribution of donations. This will allow us to closely examine the cost of fund administration. We will be interested in the costs to solicit, maintain, and track donations, offer recognition (process and materials), identify YPAO providers, and distribute funds to YPAO providers. Information from donors and YPAO providers will include name, location, contact information, and a detailed description of the donation (given or received). Because donations could be monetary (US dollars) and nonmonetary (goods/services and time), we will use the Statement of Financial Accounting Standards 157, Fair Value Measurements guide, to properly determine the US dollar value of all donation types. By the end of May 2019, the development of the beta-i was complete.

### Procedures for Aim 2

The timeline of activities involved in aim 2 is presented in Multimedia Appendix 3. During May and June, we completed baseline assessments on small businesses and YPAOs. The beta-i and the control intervention will then be implemented from July 2019 to June 2020. Follow-up assessments, which will mirror baseline assessments, will be conducted from July to August 2020. The project completion date is set for the end of August 2020.

#### Business Surveys

We developed the small business policy survey to capture the presence and development of small business support for YPAOs [67]. Using a test-retest design, we found high reliability coefficients (all>.95) for questions about YPAOs (eg, number supported and type), other physical activity promotion policies, and business/owner characteristics. In-person surveys were completed by trained personnel with a randomly selected sample of 244 small business owners. If an owner was not available, a supervisor or manager was interviewed if they indicated having the knowledge to answer our questions. We attempted to gather detailed information about the business (eg, marketing budget), the owner (eg, sex), and involvement with neighborhood initiatives. They were asked if they supported YPAOs and the cost, location, and reason for each YPAO supported. In the follow-up surveys, we will also include a series of open-ended questions to elicit information about the intervention and for the control businesses, questions about possible exposure to beta-i components and if this had influenced their YPAO-supporting activities. A thematic analysis, similar to the process outlined for the focus groups, will be done to analyze these short answer questions.

#### Youth Physical Activity Opportunity Provider Surveys

In-person interviews were conducted by trained personnel with a randomly selected sample of 44 YPAO providers. Our reliable YPAO survey was used to gather detailed information on the YPAO including the number of youth participants, descriptions of all features and amenities, programmatic information (eg, fees, operating times, and sessions per week), personnel qualifications, and start-up and operating costs including how costs were covered [52]. If a small business providing a YPAO was selected for the survey, we completed the YPAO survey with them in addition to the small business survey.

#### Interventions

As stated previously, the treatment intervention (beta-i) will be a derivative of the alpha-i intervention components given in Multimedia Appendix 2. The control intervention is based on the finding that under normal circumstances, small business owners are seldom asked to provide support for specific initiatives in their neighborhood and they almost never receive educational material about the benefits of supporting neighborhood initiatives. Usually they are just made aware of organizations (eg, nonprofits) accepting donations [67]. In keeping with this *standard of practice*, small businesses in the control neighborhoods will be offered a minimal intervention with an opportunity to donate to a fund supporting YPAOs. This fund will be established at our institution for credibility and tracking purposes. However, donors to the control fund will not be able to select specific YPAOs to support, donate directly to their neighborhood, or receive recognition for their donations. In addition, the email messages they receive will not utilize a marketing strategy and only contain basic information for donating along with contact information if they have any questions. A local advisory board will not be formed, and liaisons will not be used in the control neighborhoods. Email distributions in the control neighborhoods will coincide with those made in the treatment neighborhoods. Donations to the control fund will be given to groups not operating in the study neighborhoods to further their mission to provide the youth with low-cost obesity treatment options, such as YPAOs. Provided in Multimedia Appendix 4 is a description of the interventions.

### Procedures for Aim 3

Data for aim 3 will be collected at baseline and follow-up as per the measures described under aim 2 as well as additional measures described below and used to address our aim 3 hypothesis. These additional measures will be conducted at baseline and follow-up.

#### The System for Observing Play and Recreation in Communities

The system for observing play and recreation in communities (SOPARC) will be used according to the established protocols to count the number of youths using public YPAOs [86,87]. Briefly, each public YPAO will be visited by a trained observer who will first locate and map the size, location, and boundaries of all potential areas for leisure-time physical activity (ie, target areas). Then they will perform scans (ie, observation sweeps moving from left to right) of the target areas to obtain the desired information (eg, number and age group). Separate scans will be conducted for females and males. All parks will be assessed at baseline and follow-up 4 times a day on 4 separate days. The daily observation periods will be 7 am to 9 am, 11 am to 1 pm, 3 pm to 5 pm, and 7 pm to 9 pm and the 4 days will consist of 3 weekdays and 1 weekend day. This number of observations is the minimum needed to obtain robust estimates of park user characteristics [87]. Target areas will be assessed during each observation period according to a pre-established order determined by randomization and counterbalancing. During periods of moderate to severe precipitation, observations will be postponed until a later date that corresponds to the cancelled day/time period.

#### Physical Activity Resource Assessment

Trained field coders will use the Physical Activity Resource Assessment (PARA) to conduct concise (10-30 min) audits of YPAOs where surveys and SOPARCs were completed. The PARA is a reliable (ρ >.77) instrument for assessing characteristics of publicly available physical activity resources including YPAO [50]. It will be used to gain further insight about the quantity and quality of YPAO amenities, features, and incivilities.

#### Training

Interviewers were trained according to the guidelines developed by The Gallop Organization [88]. Several procedures were used to assure the quality of data collected. Among these were attempts at participant maximization (eg, short introductions), refusal prevention and conversion training, and other quality control functions, such as maintenance of confidentiality, monitoring of interviewers’ work, and validation of surveys to ensure respondents had actually been surveyed. Because the number of contact attempts and the patterns of businesses/YPAO operation hours are key factors impacting response rates, 2 attempts were made during each of the following periods: weekday mornings, afternoons, and nights; weekend mornings and afternoons. For the SOPARC and PARA, 2 observers/raters participated in a training session where they were given detailed instructions on the techniques [50,86].

### Analysis

#### Process Evaluations

Process outcomes will inform us of intervention challenges and lessons learned. They will be derived from meeting minutes, debriefing sessions, study records (eg, budgets), small business surveys, YPAO provider reports, adult local advisory board member reports, liaisons, and study team evaluations. The primary process outcomes will be related to the local advisory board (eg, attendance), cost of fund administration and logistics of tracking donations and providing recognition, user satisfaction with donating methods, logistics of using electronic messaging (eg, span blockers), and recruitment/retention of liaisons. This information will be used to inform planning of future implementations of the intervention and when interpreting/discussing outcomes from this study.

#### Qualitative Data Analysis Plan

An iterative, 2-phase thematic analysis will be conducted to capture the meaning behind the transcribed text with an overall purpose of creating an increasingly sophisticated and rich description about small business involvement with YPAOs. First, researchers will review the transcribed documents to develop a familiarity with the text and search for the patterns and the themes that occur frequently in a single session or are common across sessions. The data will then be coded by identifying passages that exemplify key concepts or ideas related to the major patterns and themes. The use of multiple reviewers will help establish construct validity and interrater reliability of the coding scheme and identified codes. During the second phase, a computer-assisted qualitative data analysis program (NVivo qualitative data analysis software; QSR International Pty Ltd. Version 12, 2018) will be used to check the rigor of the manual coding, help organize the large volume of data, analyze data, and provide a means for generating reports. Qualitative data analysis is typically iterative, recursive, and dynamic; therefore, we will move between the manual and the electronic process until we are satisfied that the coding scheme and results are representative of the participants’ perspectives. Furthermore, themes identified will be compared with the extant literature on the topic to further validate the findings [89].

#### Cost Analysis

The detailed information collected about YPAOs will be used to estimate the relative contributions made by small businesses toward their total yearly costs. Total yearly costs will be defined as all costs in the cost model incurred for the YPAO during the past 12 months (Multimedia Appendix 5). No discount rate will be considered for this short period. Cost-effectiveness analyses will be used to describe the total yearly costs incurred by small businesses for supporting YPAOs relative to the number of youths utilizing the YPAOs. Both 1-way and multiple way sensitivity analyses will be performed to measure the robustness of the evaluation.

#### Power Analysis

Our power analysis was based on the percent change in the proportion of small businesses supporting YPAOs at baseline that subsequently provided support for YPAOs during the intervention. Data to construct the primary outcome will be derived from tracking donations and small business surveys. Power formulas accounted for experimental condition, number of neighborhoods, number of businesses/neighborhoods surveyed, variance estimates of the outcome measure from preliminary studies, and a conservative estimate of the intraclass correlation coefficient (ICC). In our previous research, a 27% versus 8.0% increase in support for community physical activity initiatives by small businesses not supporting them at baseline was observed in the treatment and the control neighborhoods, respectively [78]. On the basis of our previous experience conducting research in neighborhoods and our desire to develop a sufficiently rich and diverse dataset, we tested whether 10 neighborhoods per condition would also provide sufficient statistical power for our primary outcome. Thus, the final power model was based on 10 neighborhoods per condition, 10 small businesses nested in each neighborhood, a difference in proportions of 19 percentage points, and an ICC of 0.05. It was determined that having 10 neighborhoods per condition would give us a statistical power of 86% to yield a statistically significant result. In a previous study, we completed interviews at 66.2% (98/148) of the small businesses visited [67]. Of them, 30.4% (45/148) refused and interviews could not be completed with an eligible individual at 3.4% (5/148) of businesses. For the proposed study, we expect an average of 50 small businesses per neighborhood or 500 per condition. Therefore, we will obtain a random sample at baseline of 185 small businesses per condition and expect to complete a baseline survey at 66.2% (122/185) per condition. Of these 122, we expect a loss-to-follow-up of 15% to 17%, giving us 104 businesses per condition or ~10 per neighborhood. This will allow us to achieve our goal of interviewing no less than 20% of the eligible businesses and obtaining a representative sample [84].

#### Approach to Analysis

Before developing statistical models, an examination of the univariate distribution of variables will be conducted (eg, scatter plots). Statistics, such as means or proportions, standard errors, ranges, and estimates of skewness and kurtosis will be derived for the overall samples and stratified by condition and characteristics of small businesses and YPAOs using SAS 9.4 and used as guidelines in the application of both bivariate and multivariate analyses [85]. Data transformation procedures (eg, logarithmic) may be applied to quantitative variables whose distribution shows considerable departure from normality. In the case of discrete variables, results will provide guidance in recoding these variables appropriately for statistical modeling. Graphical data will be developed to provide visual comparisons of changes across time between the 2 study conditions on key measures of small business involvement and YPAO utilization by the youth. The primary outcome will be examined using a mixed-model, nested logistic regression analysis of proportions where (1) businesses are nested in neighborhoods and (2) neighborhoods are treated as a nested random effect within treatment conditions. Generalized models will be used to explore potential mediators/moderators of outcomes. For example, a model will be constructed with experimental condition (treatment vs control), neighborhood variables (eg, median income), and business variables (eg, number of employees and years in business) as the independent variables and mean percent change in small businesses donating as the dependent variable. In another model, donation amounts equated to US dollars will be used as the dependent variable. To accommodate the complex nature of the research design, the SAS PROC GLIMMIX and SAS PROC MIXED (SAS, 2015) procedures will be used.

### Sample Size Determination for Aim 3

The primary outcome for aim 3 will be the percent increase in youth participants from baseline to follow-up. We hypothesize that the increase will be significantly greater at YPAOs in the treatment neighborhoods receiving support from small businesses than at YPAOs in the control neighborhoods. Previously in low-income, minority neighborhoods, we found an average of 46.7 (SD 37) youths participating in YPAOs [52,90]. With the proposed sample size of 10 neighborhoods per condition from aim 2, our pilot parameter estimates, an ICC of 0.05, and a desired power of at least 80%, we would need 35 YPAO assessments per condition to detect a difference of 0.5 percentage points between conditions at posttest. We expect a total of 140 YPAOs in our 20 study neighborhoods with 20% (28/140) being eligible for SOPARC assessments [52]. Given a 15% to 17% attrition rate and a response rate of 60%, we will randomly select 90 YPAOs for interviews to yield 44 YPAOs with baseline and follow-up data from the survey. These will be combined with the 28 YPAOs observed, giving a total of 70.

### Potential Problems and Solutions

#### Attrition and Evaluation of Missing Data

Over the past 2 decades, our research team has developed methods to ensure low rates of missing data in our projects. One aspect of this study most likely to result in missing data is the longitudinal assessment of businesses/YPAOs. We will attempt to minimize missing data by maintaining contact with study businesses/YPAO providers and if dropout does occur, we will attempt to determine the reasons why and how much of their study participation was affected. We will examine characteristics associated with attrition and adjust models for attrition and/or baseline group differences.

#### Appealing to Small Businesses

We have been successful in the past in obtaining information from small businesses and soliciting their support for community physical activity initiatives. In the proposed study, we expect to elicit significant increases in support for YPAOs from small businesses in the treatment neighborhoods because the intervention contains numerous components that stimulate donations from small businesses.

#### Measuring Youth Physical Activity Opportunity Utilization

We do not foresee a problem recruiting YPAO providers to participate in the study. We were successful at obtaining detailed information from YPAO providers in a previous study and, in the proposed study, they will receive compensation for their participation and have the opportunity to receive donated funds [52].

## Results

The study protocol was approved by our institutional review board on July 13, 2018. All preparatory activities (eg, hiring, training, and data collection procedures finalized) were completed on 31 October 2018. In addition, 20 study neighborhoods comprising 53 US census block groups meeting the inclusion criteria were identified and randomly assigned to treatment or control conditions (Multimedia Appendix 6). A total of 9 focus groups were completed with members from the study neighborhoods who were small business owners, YPAO providers, or parents and guardians. Recruitment was accomplished in a number of different ways—requests made at events (eg, business chamber meeting), business chamber networking, direct contact (eg, email, phone, and site visits), ad placements, and active recruitment at community locations. Focus group data have been transcribed and are currently being analyzed. It will be utilized in May 2019 to revise any components of the intervention and expected results will be published in fall 2019.

In December 2018, a database containing the names, addresses, and geolocations (eg, US census block group) of the 87,982 businesses currently licensed in Delaware was obtained from the Delaware Department of Finance: Division of Revenue. The database was edited to include only small businesses with a physical, nonresidential address located in 1 of our 20 study neighborhoods. During May and June, 2019, baseline surveys were completed with 244 owners and managers of small businesses in the study neighborhoods. Data from small businesses will be analyzed in July 2019 and results will be disseminated in August 2019. In addition, a total of 96 YPAOs were identified in the 20 study neighborhoods, and interviews have been conducted with 35 nonpark YPAOs from May 6 to June, 2019. The park YPAOs (n=27) were examined in June, 2019 using the SOPARC and PARA methods to determine usages and presence/absence of amenities.

## Discussion

We are 10 months into this study and have achieved the milestones set forth in the proposal. Preparation activities, including the identification of study neighborhoods and small businesses and YPAOs in these neighborhoods, and focus groups have been completed. The focus group data are currently being analyzed and we have just completed (June 2019) baseline surveying of small business owners and managers and YPAO providers. It is anticipated that findings will be disseminated in the fall of 2019.

Research in this area has typically been on policies targeting employee wellness programs at large corporations [91]. The proposed study will be the first to generate evidence on changing small business policies to mobilize their resources for YPAOs by applying previously proven strategies for stimulating support of community initiatives in a novel way. To our knowledge, we are the only group attempting to understand how the power of small businesses can be harnessed to promote healthy lifestyles in the youth. This effort will generate new knowledge about the alternative sources of support (eg, private sector) for YPAOs. Most existing descriptions of community-level physical activity interventions focus on support emanating primarily from governments and government-based institutions, such as public schools [92,93]. Increasing YPAO support from small businesses could result in a shift (reduction) in resource responsibility for youth physical activity promotion from these more traditional sources. A reduction in support from government-related entities may actually stimulate private giving for community initiatives [94,95]. Economists, public health personnel, and government officials would view such a shift as an improvement in the use of resources, as well as a cost-effective method for providing sustainable interventions to promote health [75,96]. Furthermore, having additional funds for health promotion in low-income areas opens the door for implementing novel approaches using the latest technology (eg, 3-dimensional printed physical activity models) and mobile health apps [97,98].

## Appendix

Multimedia Appendix 1 Aim 1 activities.

Multimedia Appendix 2 Intervention alpha components.

Multimedia Appendix 3 Aim 2 activities.

Multimedia Appendix 4 Description of treatment and control interventions.

Multimedia Appendix 5 Cost model.

Multimedia Appendix 6 Characteristics of study neighborhoods [means (standard deviations)].

## Abbreviations

alpha-i: alpha version of intervention
beta-i: beta version of intervention
ICC: intraclass correlation coefficient
MVPA: moderate to vigorous physical activity
PARA: Physical Activity Resource Assessment
PE: physical education
SOPARC: system for observing play and recreation in communities
YPAO: youth physical activity opportunity
