# Supplementary figures and images for: Promoting Small Business Support of Youth Physical Activity in Low-Income, Minority Neighborhoods: Protocol for a Randomized Controlled Trial
Source: JMIR Res Protoc. 2019 Jul 30;8(7):e13141. doi: 10.2196/13141 (PMC6691677; doi:10.2196/13141)

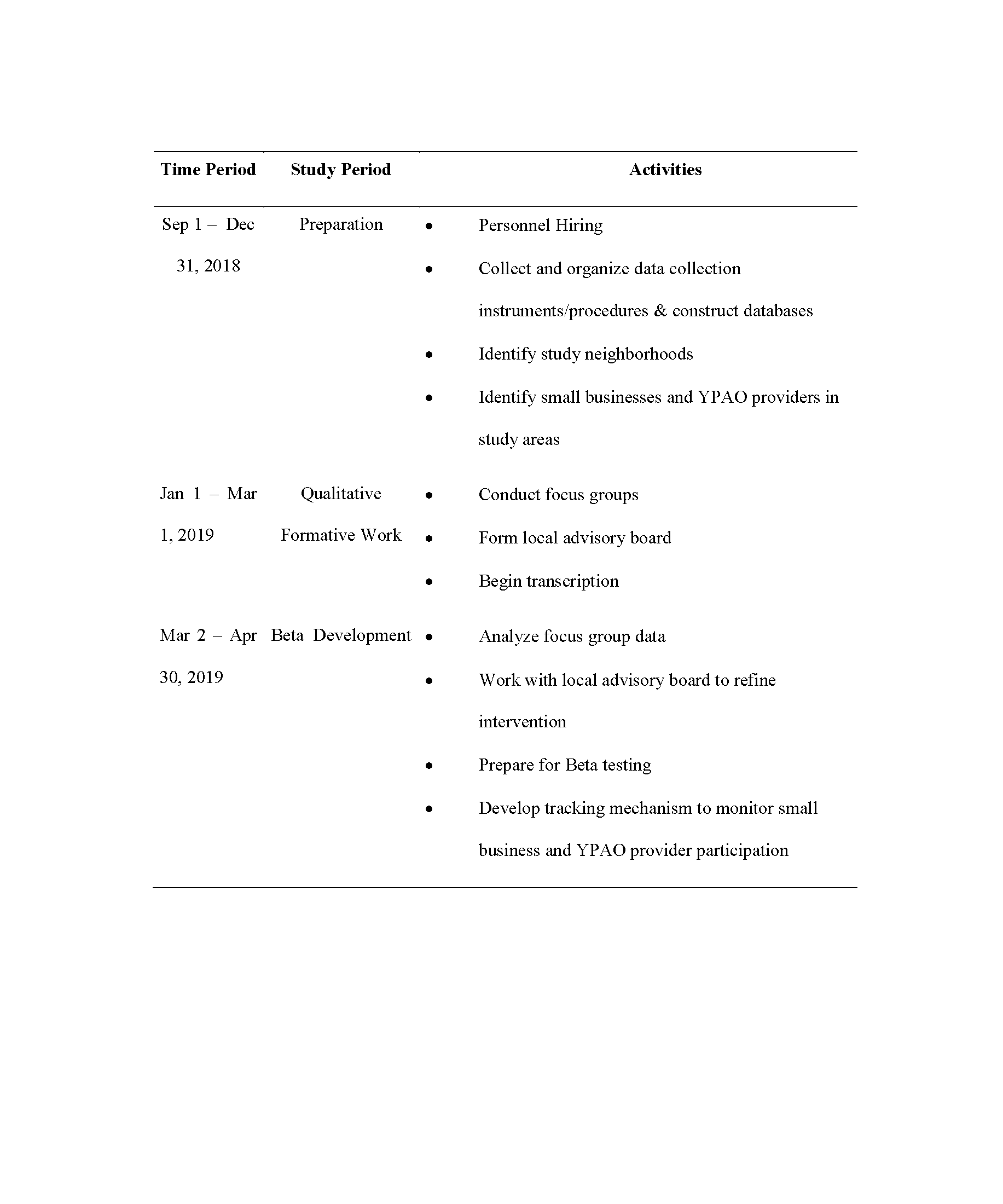

Supplement: Multimedia Appendix 1 [file resprot_v8i7e13141_app1.png]

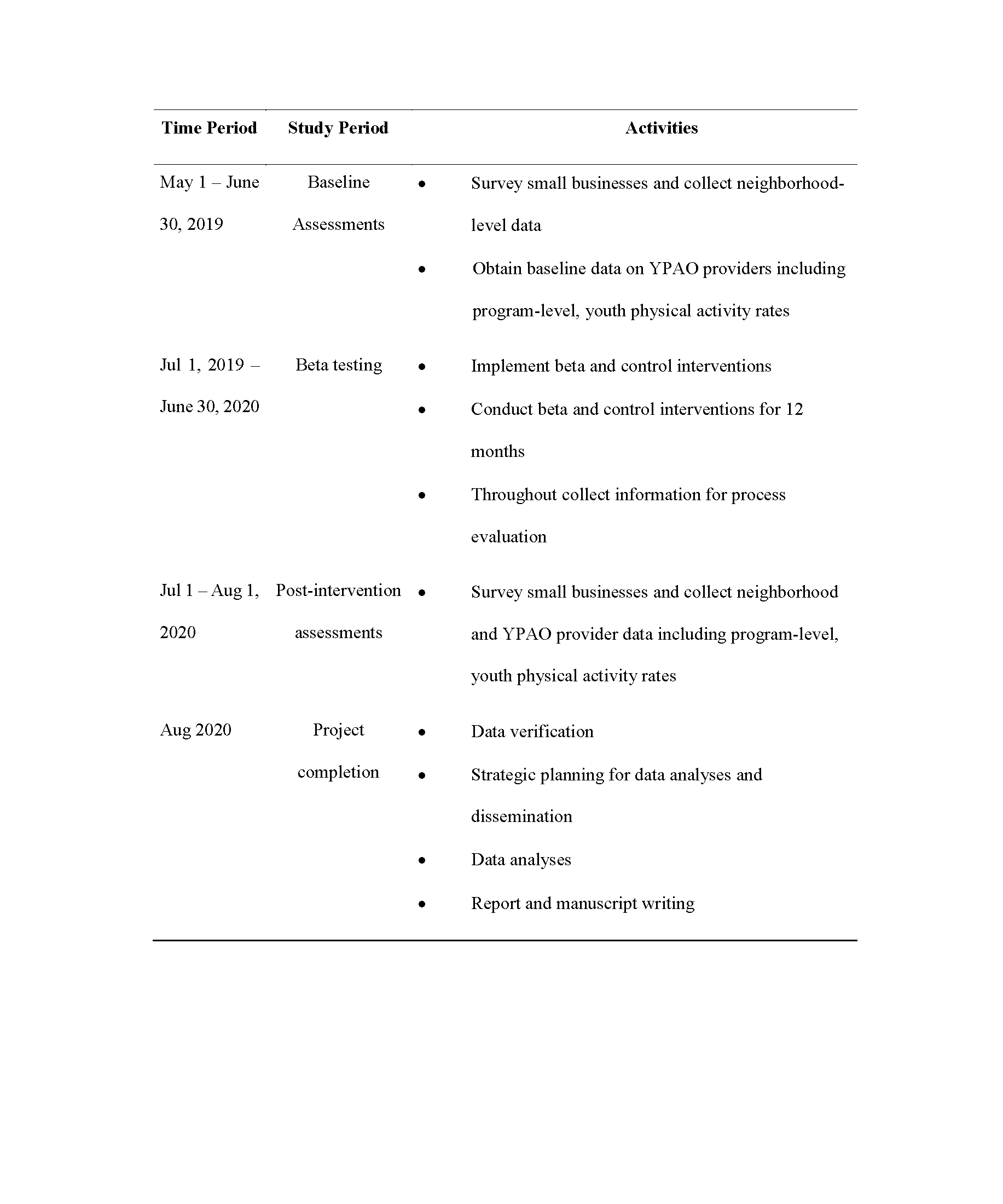

Supplement: Multimedia Appendix 3 [file resprot_v8i7e13141_app3.png]

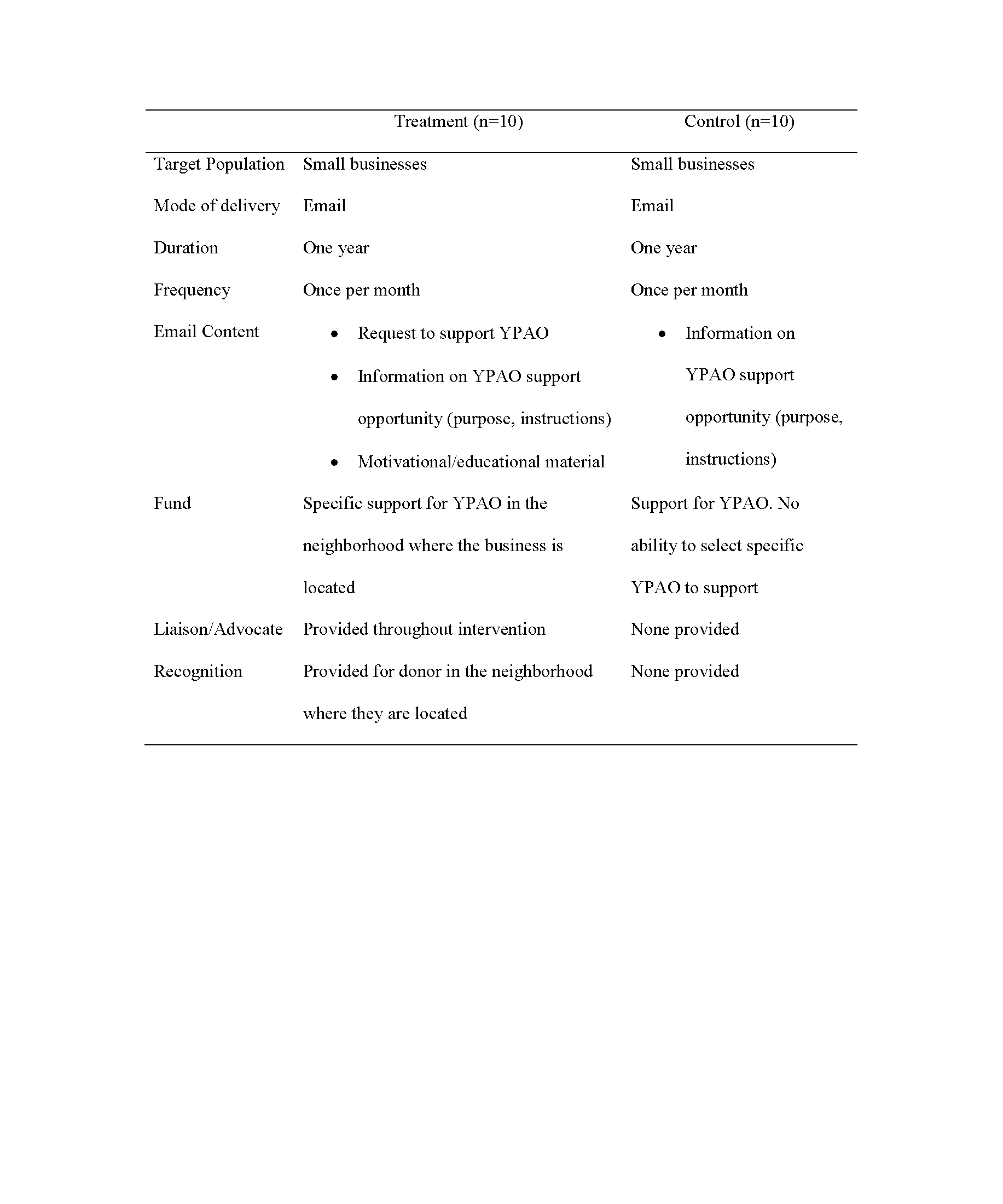

Supplement: Multimedia Appendix 4 [file resprot_v8i7e13141_app4.png]

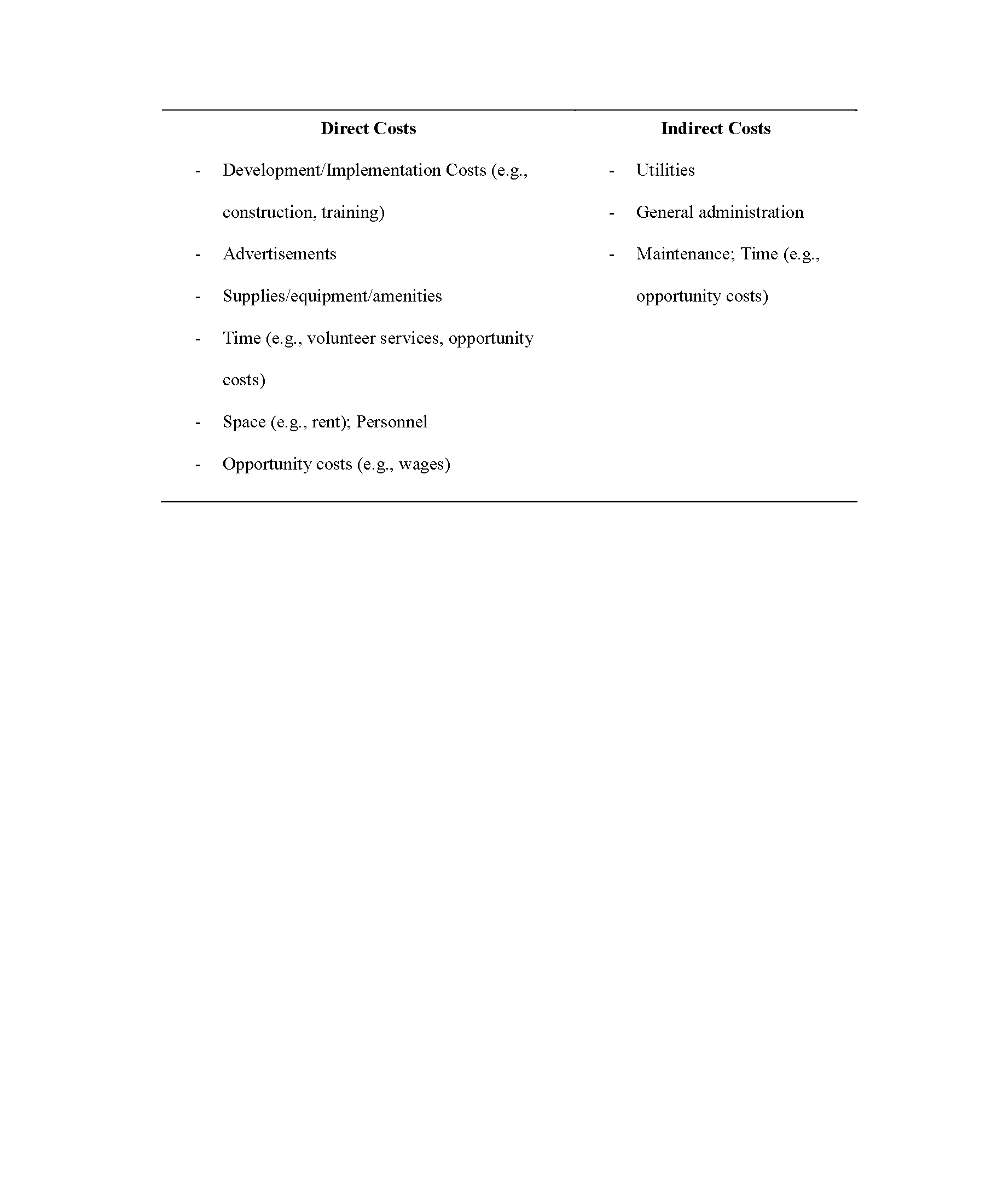

Supplement: Multimedia Appendix 5 [file resprot_v8i7e13141_app5.png]

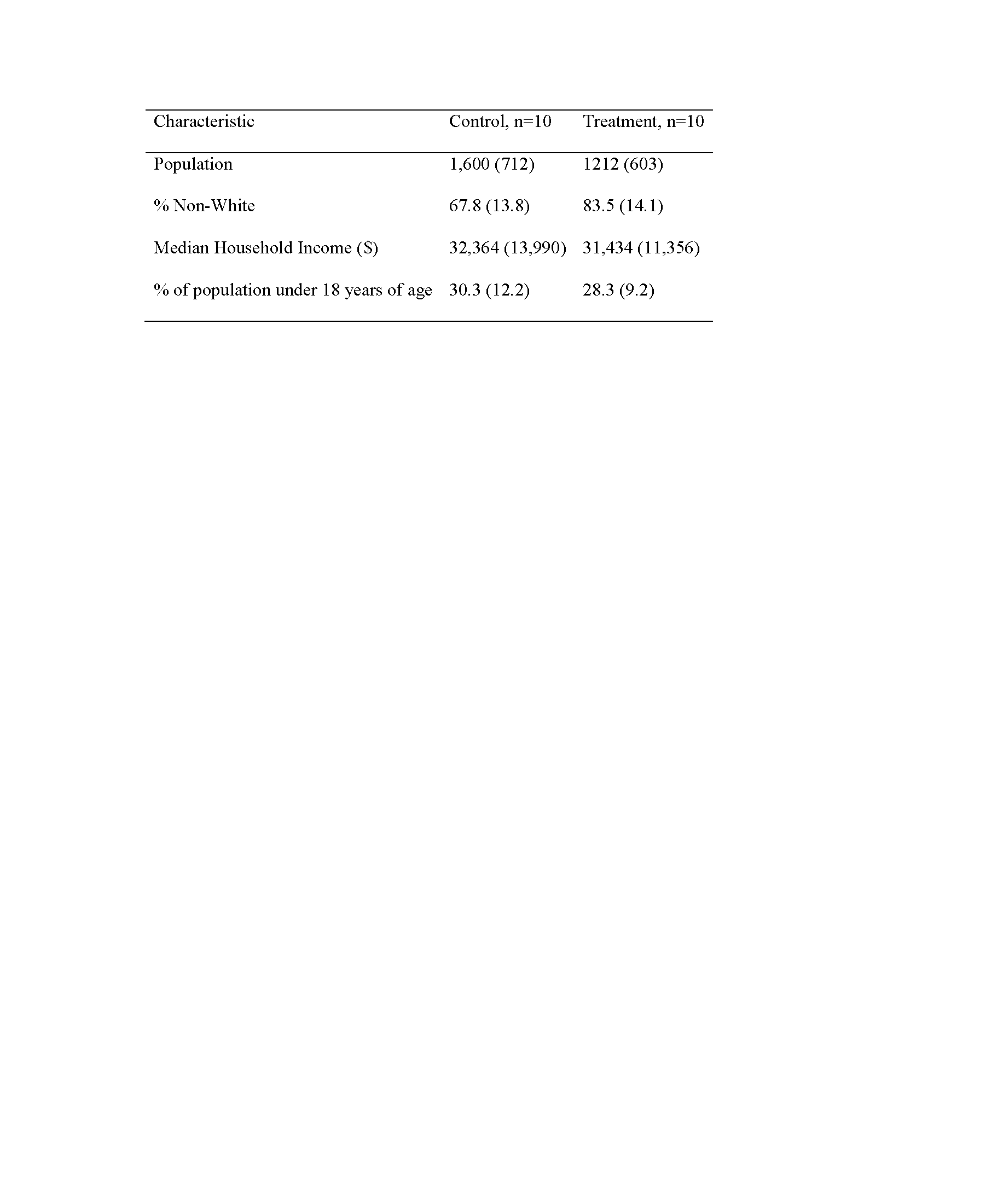

Supplement: Multimedia Appendix 6 [file resprot_v8i7e13141_app6.png]
