# Supplementary material for: Promoting Small Business Support of Youth Physical Activity in Low-Income, Minority Neighborhoods: Protocol for a Randomized Controlled Trial
Source: JMIR Res Protoc. 2019 Jul 30;8(7):e13141. doi: 10.2196/13141 (PMC6691677; doi:10.2196/13141)
Supplement: Multimedia Appendix 2 [file resprot_v8i7e13141_app2.pdf]

Alpha-i Components

Local Advisory Board (LAB): One LAB will be formed for the treatment neighborhoods. Its responsibilities will be to examine focus group data and help create the beta-i, provide feedback on data collection and process outcomes, make YPAO funding decisions, and help interpret study findings. Prior to the completion of the focus groups, 10 LAB members will be recruited from different neighborhood sectors, include one individual from each treatment neighborhood, and include one to two adolescents.

Delivery: Email will be used as the primary means of communicating with small businesses. Email messages will be sent monthly and contain a request to provide support for YPAOs along with a list of YPAOs located in the neighborhood, motivational/educational information including success stories, the names of business donors, and detailed information about the campaign. We are using email because it's a low-cost communication method for reaching large populations, it can facilitate the giving process, and interventions delivered via email promote changes in behavior [88,89]. In our past studies, we were always able to find a person at a business involved with philanthropic choices and 95% of the time we were able to obtained an email for this person [71,73]. In a pilot project, we found that 75% of the small businesses we sent an email to asking for a reply did so within a week of our request. Therefore, locating small businesses, obtaining emails for the appropriate contact person, and having them open and read the email will not be a limitation.

Fund: A fund will be established for the sole purpose of supporting YPAOs in the treatment neighborhoods. Monetary and non-monetary (e.g., goods/services, time) resources will be accepted. The process of donating to the fund will be structured to facilitate the giving process by small businesses, allow them to direct their donations to YPAOs in their neighborhood, and

provide us with the ability to track donations [89-93]. Donations will be solicited using a marketing strategy that employs proven nonprofit direct marketing tactics, including personalized email messages touting the benefits of contributing to the fund and the positive impacts resulting from donations [68, 90-97]. The email messages will include detailed directions on how to donate to the fund. Options for making donations may include: 1) use of a secure donation website with PayPal or credit card options; 2) check submission via mail; and/or 3) contacting project staff (e.g., liaison) and making arrangements for conveying donations. The fund will be established at our institution to enhance credibility and realism.

Solicitation: Email messages will contain a statement requesting donations be made to the fund in the name of the business. Research indicates this is a critical action for moving small businesses from non-YPAO to YPAO supporters [71,91,97].

Motivational information: When small businesses are notified of the fund and asked for donations, literature will be included clearly expressing the mission of the campaign, the importance of YPAOs, and the benefits of supporting YPAOs. In essence, this is our sales pitch. Some of the information to be highlighted includes: Good will/importance (give back to your local neighborhood, enhance the development and health of youth, improve local neighborhood conditions and increased revenue (improve your business image which increases customers, receive free local advertising - majority of customers of small businesses are from the local area, and lower taxable income [63,66,68,92,93].

Recognition: All non-anonymous, small business donors will be recognized for their contributions [94,95]. Recognition will be focused in the neighborhood where the donor is located, provided in monthly emails, and economical (signs, website postings). During this study, recognition costs will be covered by grant funds; however, we will closely monitor this cost to determine the actual

expense of providing recognition. This will help inform applications of the intervention beyond this study where recognition costs may be covered by donations.

Neighborhood liaisons: It is important to establish a relationship between the donor and the recipient organization [93]. We found that small business owners who supported YPAOs were more likely to know someone affiliated with the YPAO [71]. Therefore, we will recruit two residents who are YPAO providers from each treatment neighborhood to volunteer as liaisons. They will visit each small business in their neighborhood before the business receives its first email and 6-months later. Liaisons will be available to businesses throughout the intervention (via phone, email, text messaging).

Promotion of fund use: YPAO providers in the treatment neighborhoods will be notified about the funds and given instructions on how to apply. The process will involve the submission of a concise proposal to the LAB describing the YPAO and how funds will be used. Funds will be awarded after a proposal is reviewed and the authenticity of the YPAO verified (e.g., site visit). Any further requirements including award amounts will be determined by the LAB.
